# Functional outcome, five-year survival and burden of disease after size- and location-matched hemorrhagic versus ischemic stroke

**DOI:** 10.1186/s42466-026-00456-w

**Published:** 2026-02-03

**Authors:** Stefanie Balk, Teresa Siller, Maximilian I. Sprügel, David Haupenthal, Kathrin Kölbl, Stefan Hock, Daniel Heinze, Tobias Engelhorn, Bernd Kallmünzer, Stefan Schwab, Hagen B. Huttner, Joji B. Kuramatsu, Jochen A. Sembill

**Affiliations:** 1 Department of Neurology, University Hospital Erlangen, Schwabachanlage 6, 91054 Erlangen, Germany; 2 Department of Neurology, University Hospital Bern, Freiburgstrasse 16, 3010 Bern, Switzerland; 3 Department of Neuroradiology, University Hospital Erlangen, Schwabachanlage 6, 91054 Erlangen, Germany; 4 Department of Neurology, Carl Gustav Carus University Hospital Dresden, Fetscherstraße 74, 01307 Dresden, Germany; 5 Department of Neurology, Community Hospital RoMed Klinikum Rosenheim, Ellmaierstraße 23, 83022 Rosenheim, Germany

**Keywords:** Ischemic stroke, Hemorrhagic stroke, Functional outcome, Lesion size, Disability-adjusted life years

## Abstract

**Background:**

Worse functional outcome has been reported after hemorrhagic compared to ischemic stroke, yet it remains unclear whether the lesion size, location, or stroke etiology itself results in different degrees of disability. This study compares outcomes in ischemic vs. hemorrhagic stroke patients with matched lesion size and location.

**Methods:**

Data from the longitudinal cohort study on intracerebral hemorrhage (ICH) care (2006–2015) and a retrospective registry on acute ischemic stroke (AIS) (2011–2015), both conducted at the university hospital Erlangen, were used to investigate patients with a single stroke lesion in the middle cerebral artery territory. Patient characteristics, lesion size and affected brain location according to the Alberta Stroke Program Early CT Score (ASPECTS) regions were balanced using propensity score matching. Functional outcomes (ordinal shift of modified Rankin scale (mRS) at 3 months), 5-year survival (Kaplan–Meier, Cox regression), and burden of disease (Disability-Adjusted Life Years [DALYs] = Years of Life Lost [YLL] + Years Lived with Disability [YLD]) were compared.

**Results:**

After propensity score matching, we analyzed 194 patients, divided equally into AIS or ICH cases. We observed a shift in mRS distribution towards better functional outcomes in favor of AIS over ICH (OR: 1.69, 95% CI: 1.02–2.79, p = 0.04), especially in younger patients (age < 75 years, OR: 3.23, 95% CI: 1.58–6.59, p < 0.01) and smaller lesion volumes (< 15 mL, OR: 4.48, 95% CI: 1.85–10.89, p < 0.01). The 5-year survival did not differ between groups (log-rank 0.63). Mean number of DALYs was higher following ICH compared to AIS in general (8.39 ± 5.18 vs. 5.90 ± 4.61, p < 0.001) and per milliliter of parenchymal lesion volume (0.31[0.14–0.81] vs. 0.24[0.10–0.41], p = 0.04). The higher number of DALYs is attributable to the higher number of YLDs (ICH, 3.49 ± 3.3 vs. AIS, 2.31 ± 2.86, p < 0.01) and YLLs (ICH, 4.90 ± 3.27 vs. AIS, 3.59 ± 3.08, p < 0.01) in matched patients following ICH compared to AIS.

**Conclusions:**

For stroke lesions of matched size and location, patients with hemorrhagic stroke had worse functional outcome, greater disease burden in total and per ml, yet comparable long-term survival rates compared to those with ischemic stroke.

## Introduction

Worldwide, stroke is the second most common cause of death and the most common cause of disability [1]. Patients who have suffered a stroke have worse outcomes with 3-month mortality rates of 35–65% after intracerebral hemorrhage (ICH) versus 15–25% after acute ischemic stroke (AIS), whereby the functional outcome is also observed to be more favorable after AIS [2–5]. However, some studies have described equal or even greater progress in rehabilitation after ICH than after AIS [6–9]. Studies comparing the impact of ICH vs. AIS have so far considered the clinical stroke severity and other factors such as age and co-morbidities, but precise matching with regard to specific imaging criteria of the stroke lesion has not yet been sufficiently performed [5, 6, 8, 10–12]. In addition, information on long-term survival and the burden of living with disability for an individual’s remaining life comparing these two entities would be valuable to inform public health strategies [13]. This comparative observational study is the first study of its kind to compare the functional outcome after 3 months, the 5-year overall survival and the burden of disease measured by DALYs in a defined cohort of patients with hemorrhagic and ischemic stroke lesions matched by size and location on imaging.

## Methods

### Study design and participants

We merged data from two different cohort studies conducted at the University Hospital Erlangen, Germany. For patients with AIS, we used the retrospective institutional registry that included patients with AIS who received intravenous (IV) thrombolysis or thrombectomy between 2011 and 2015 [14, 15]. For patients with ICH, we used the Longitudinal Cohort Study on ICH Care (UKER-ICH, NCT03183167, registration date 2017–06-08) including patients with spontaneous ICH between 2006 and 2015 [16, 17]. To achieve the best possible comparability, we only included patients with a hemorrhagic or ischemic stroke lesion in the middle cerebral artery (MCA) territory. Our exclusion criteria were designed to minimize confounding of patients’ outcomes beyond the specific stroke lesions and comprised patients with multiple stroke lesions or new stroke lesions or symptoms during the course, ICH secondary to ischemic stroke, ICH with intraventricular extension or hematoma expansion, and patients requiring brain surgery [18, 19].

### Data collection

Demographic data, including clinical characteristics, premorbid conditions, and in-hospital parameters, were assessed through review of patients’ medical records and institutional databases, as previously published [14, 16, 20, 21]. Follow-up data were collected by trained raters via mailed questionnaires or semiquantitative telephone interviews, outpatient visits, medical reports, or retrieved from institutional databases in case of hospital readmission, as previously described [13, 14]. Functional outcome was evaluated using the modified Rankin scale (mRS), ranging from no symptoms (0) to severe disability (5) and death (6) [13, 14].

### Imaging acquisition and analysis

**Fig. 1:**
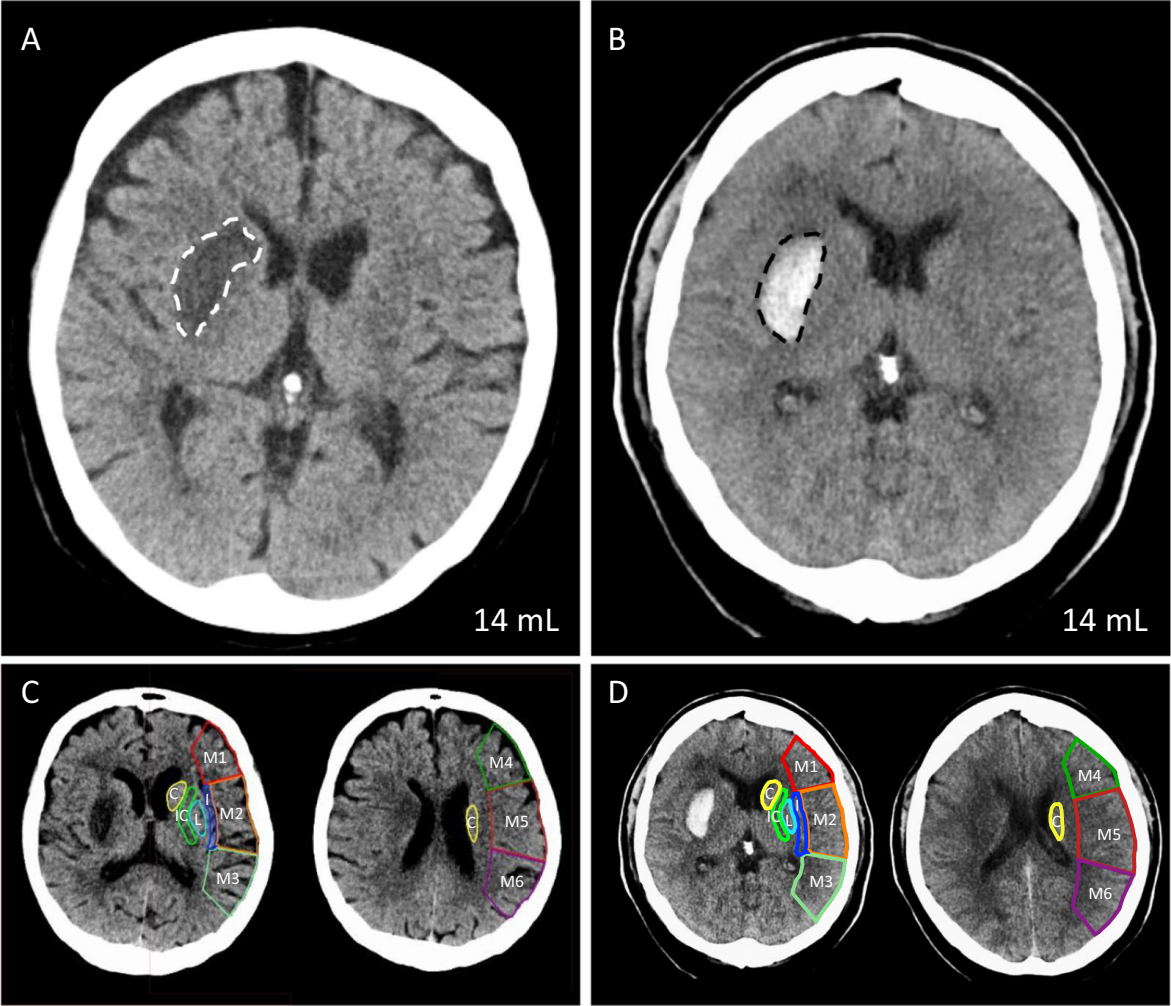
Methodology for imaging-based assessment of stroke lesions. This figure illustrates how comparable stroke topography was ensured using propensity score matching. Shown is a matched pair of stroke lesions on non-contrast CT: One AIS (A, white dashed line) and one ICH (B, black dashed line) with comparable lesion volume (14 mL) and similar anatomical location based on the ASPECTS. The corresponding ASPECTS regions are illustrated on the contralateral side for the AIS (C) and the ICH (D). C, Caudate; I, insular cortex; IC, internal capsule; L, lentiform nucleus; M1, anterior MCA cortex, which corresponds to the frontal operculum; M2, MCA cortex lateral to the insular ligament, corresponding to the anterior temporal lobe; M3, posterior MCA cortex, corresponding to the posterior temporal lobe; M4, anterior MCA territory immediately superior to M1; M5, lateral MCA territory immediately superior to M2; M6, posterior MCA territory immediately superior to M3

Diagnostic and follow-up CT scans were performed according to the standardized protocols of the institution, anticipated at 24 hours after initial imaging. The stroke size and location were determined based on the maximum lesion on the follow-up CT scan, i.e. maximum infarct size or stable hematoma volume, by 2 board certified neuroradiologists in consensus [22]. To determine the final lesion size of the infarct or ICH, we used a validated semi-automated volumetric method, as previously described [23]. The localization was classified by affected areas according to the localizations used to calculate the ASPECTS, a 1 to 10-point topographic CT scan score used for MCA stroke patients, including caudate, lentiform nucleus, internal capsule, insular cortex, M1: anterior MCA cortex, which corresponds to the frontal operculum, M2: MCA cortex lateral to the insular ligament, corresponding to the anterior temporal lobe, M3: posterior MCA cortex, corresponding to the posterior temporal lobe, M4: anterior MCA territory immediately superior to M1, M5: lateral MCA territory immediately superior to M2, and M6: posterior MCA territory immediately superior to M3 [24–26]. Figure 1A-D illustrates the methodology employed for imaging assessment, exemplified by CT imaging of a patient with ischemic stroke and a matched patient with a comparably localized and comparably sized hemorrhagic stroke.

### Statistics

Statistical analyses were carried out using the statistical software SPSS, version 24.0 (IBM) and Graphpad Prism, version 9 (GraphPad Software, Inc.). We applied two-sided statistical tests and set the significance level at 0.05. We report categorical variables as numbers and percentages, evaluated by the χ2 test or Fisher exact test, and ordinal and continuous variables as medians and interquartile ranges (IQR), compared by the Mann–Whitney U test. We conducted propensity score matching using a parallel, balanced, 1:1 ratio nearest-neighbor approach to adjust for significant imbalances of the cohorts in demographic and radiological baseline characteristics, i.e. sex, affected ASPECTS localizations, and maximum lesion volume [16]. For matching procedure we aggregated ASPECTS location into the anterior MCA territory, i.e. region M1 and M4, the lateral MCA territory, i.e. region M2 and M5, the posterior MCA territory, i.e. region M3 and M6, the deep MCA territory, i.e. caudate, putamen, and internal capsule, and the insular cortex. The year of treatment was not included in the matching procedure, as it indicated no association with favorable functional outcome in selected patients with either ICH (OR 0.82, CI 0.61–1.09, p = 0.37) or AIS (OR 0.99, CI 0.77–1.28, p = 0.94). We compared functional outcomes between matched cohorts and among relevant subgroups, i.e. patients classified by sex and age as well as by lesion side, location and size, using ordinal shift analyses of the mRS at 3 months. Additionally, the bivariate correlation between lesion volume and mRS was assessed using the Pearson correlation coefficient (r). We assessed the cumulative survival over 5 years of matched patients with AIS compared to ICH using Kaplan–Meier estimates and calculated corresponding hazard ratios using COX regression modelling. To assess the burden of disease, we calculated DALYs for each patient with AIS or ICH. This measure represents a time-based evaluation of health status, expressed in terms of healthy life years lost [13, 27, 28]. The calculation is based on the summation of observed Years of Life Lost (YLL) + Years Lived with Disability (YLD), i.e. DALY = YLL + YLD [13, 27]. YLL are the difference between the patient’s age-specific life expectancy and the age at which death occurs, and YLD is defined as the product of the number of years lived with disability according to most recent functional follow-up status multiplied by a disability-weighting factor, in accordance with the established classification by the World Health Organization [13, 27]. The specific disability weights were: mRS 0 0.000 mRS 1 0.046 mRS 2 0.212 mRS 3 0.331 mRS 4 0.652 mRS 5 0.944 [13, 29]. We further calculated the specific burden of disease per milliliter of parenchymal lesion and compared it between patients with AIS and ICH.

### Standard protocol approvals, registrations, and patient consents

Both studies, the retrospective institutional registry for AIS and the Longitudinal Cohort Study on ICH Care, were approved by the local institutional review board, and informed consent was obtained from patients or their legal representatives [14-17].

## Results

### Patient characteristics

**Fig. 2:**
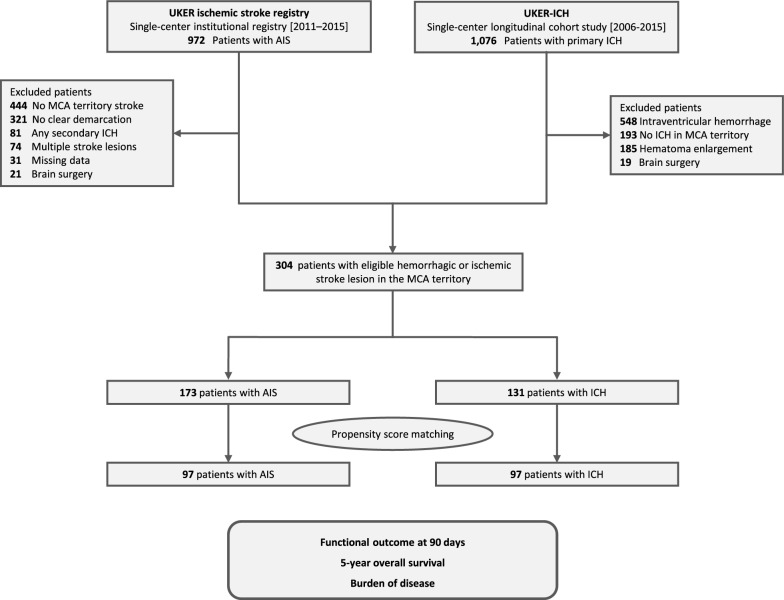
Study cohorts and matching procedure. The analysis combined two cohorts from the same center: the UKER Ischemic Stroke Registry and the Longitudinal Cohort Study on ICH Care (UKER-ICH). To ensure optimal comparability, we included only patients with stroke lesions located in the MCA territory and excluded those with multiple or non-definable lesions, secondary ICH, intraventricular hemorrhage or unstable hematoma, as well as patients who required neurosurgical intervention. Propensity score matching was applied to minimize imbalances in demographic characteristics and in the size and location of the stroke lesions. The flowchart indicates patient numbers at each selection and matching step. AIS, acute ischemic stroke; ICH, intracerebral hemorrhage; MCA, middle cerebral artery

We screened 1,076 patients with ICH and 972 patients with AIS. A total of 304 patients met the selection criteria, 131 patients with ICH and 173 patients with AIS, see Fig. 2 for study flowchart. The baseline characteristics of patients are shown in Table 1A. Patients with AIS compared to ICH were more frequently female [n = 98/173 (56.6%) vs. n = 59/131 (45.0%), p = 0.045], and had larger stroke lesions [45.0 mL (15.0–133.0) vs. 18.3 mL (5.7–37.7), p < 0.001] which resulted in higher frequency of ASPECTS localizations being affected [e.g., the insular cortex, n = 113/173 (65.3%) vs. 41/131 (31.3%), p < 0.001]. No relevant differences were identified with regard to the age of the patient or their medical history. We performed propensity score matching to control for observed differences, i.e. sex, stroke lesion size and affected location according to ASPECTS regions. We did not include the duration from symptom onset to CT with maximum lesion volume into matching procedure as we considered the median difference of 4 hours [AIS: 28 h (25–47) vs. ICH: 24 h (6–44), p = 0.003] to be of inconsequential clinical significance. This resulted in evenly balanced cohorts of 97 patients with AIS and 97 patients with ICH (Table 1B**)**.

**Table 1:** Baseline characteristics of patients with ischemic stroke vs. intracerebral hemorrhage

A) Before propensity score matching	AIS (n = 173)	ICH (n = 131)	*p*-value
Demographics			
Age, median (IQR), years	74 (64–82)	71 (64–81)	0.29
Sex (female), No. (%)	98 (56.6)	59 (45.0)	0.045
Prior medical history			
Arterial hypertension, No. (%)	147 (85.0)	116 (88.5)	0.51
Diabetes mellitus, No. (%)	51 (29.5)	36 (27.5)	0.66
Coronary heart disease, No. (%)	50 (28.9)	42 (32.1)	0.60
Hypercholesterolemia, No. (%)	56 (32.4)	60 (45.8)	0.35
Premorbid-mRS (IQR)	0 (0–2)	1 (0–2)	0.17
Imaging characteristics			
Duration from symptom onset to CT with maximum lesion volume (IQR), hours	28.9 (25.0–72.6)	23.2 (6.0–48.6)	< 0.001
Lesion localized at left hemisphere (%)	95 (54.9)	65 (49.6)	0.67
ASPECTS localizations ^a^			
Anterior MCA territory, No. (%)	85 (49.1)	17 (13.0)	< 0.001
Lateral MCA territory, No. (%)	106 (61.3)	51 (38.9)	< 0.001
Posterior MCA territory, No. (%)	70 (40.5)	47 (35.9)	0.42
Insular cortex, No. (%)	113 (65.3)	41 (31.3)	< 0.001
Deep MCA territory, No. (%)	104 (60.1)	63 (48.1)	0.024
Maximum lesion volume (IQR), mL	45.0 (15.0–133.0)	18.3 (5.7–37.7)	< 0.001
**B) After propensity score matching**	**AIS (n = 97)**	**ICH (n = 97)**	***p*-value**
Sex (female), No. (%)	50 (51.5)	53 (54.6)	0.67
Duration from symptom onset to CT with maximum lesion volume (IQR), hours	28 (25–47)	24 (6–44)	0.003
ASPECTS localizations ^a^			
Anterior MCA territory, No. (%)	25 (25.8)	17 (17.5)	0.16
Lateral MCA territory, No. (%)	43 (44.3)	43 (44.3)	1.00
Posterior MCA territory, No. (%)	29 (29.9)	28 (28.9)	0.88
Insular cortex, No. (%)	39 (40.2)	40 (41.2)	0.88
Deep MCA territory, No. (%)	45 (46.4)	50 (51.5)	0.47
Maximum lesion volume (IQR), mL	21.0 (9.0–44.4)	25.7 (7.8–46.8)	0.70

Shown are characteristics of patients with AIS compared to ICH A) before propensity score matching and B) balanced values of the matched parameters with previous differences after propensity score matching. We did not include the duration from symptom onset to CT with maximum lesion volume into matching procedure as we considered the median difference of 4 h [AIS: 28 h (25–47) vs. ICH: 24 h (6–44), p = 0.003] to be clinically irrelevant

^a^ASPECTS, range 0–10, segmental assessment of the MCA vascular territory. For matching procedure, the regions were aggregated as follows: Anterior MCA territory corresponds to region M1 and M4; Lateral MCA territory corresponds to region M2 and M5; Posterior MCA territory corresponds to region M3 and M6; Deep MCA territory corresponds to ASPECTS localizations caudate, putamen, and internal capsule. Multiple territories are possible if several regions are affected

AIS, acute ischemic stroke; ICH, intracerebral hemorrhage; IQR, Interquartile range (25th-75th percentile); MCA, middle cerebral artery

### Functional outcome

**Fig. 3:**
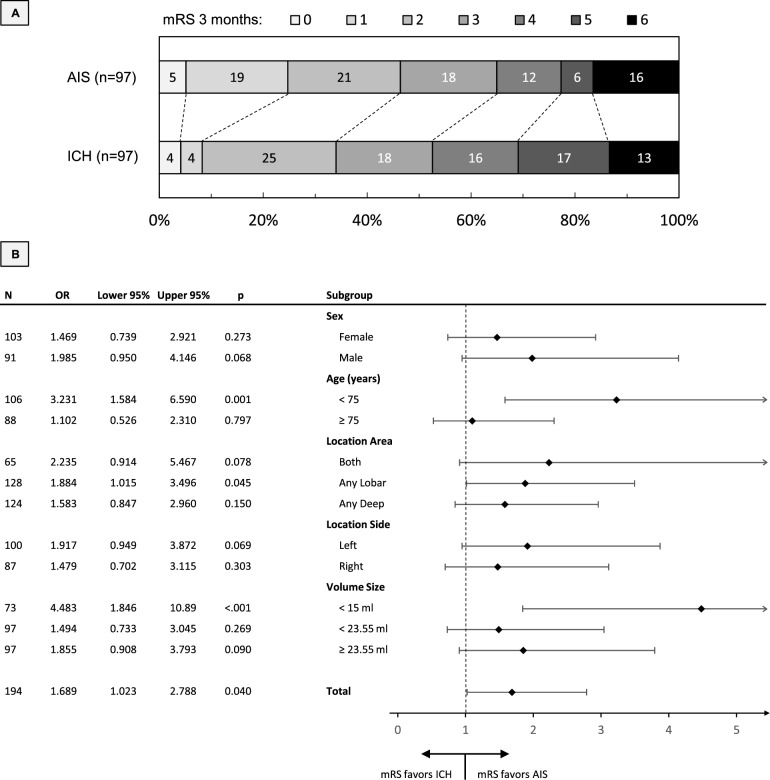
Functional outcome after 3 months. 3A: Ordinal shift analysis showing the association between stroke etiology and the mRS at 3 months. Patients with AIS demonstrated a shift toward better functional outcomes compared with ICH. 3B: Ordinal shift analysis of mRS at 3 months stratified by patient subgroups, including sex, age, affected brain regions (deep MCA territory: caudate, putamen, internal capsule, insular cortex; lobar MCA territory: M1–M6; or both), stroke hemisphere, and stroke lesion volume. AIS, acute ischemic stroke; ICH, intracerebral hemorrhage; MCA, middle cerebral artery; mRS. modified Rankin Scale

We compared functional outcomes between matched cohorts using ordinal shift analysis of the mRS at 3 months. As shown in Fig. 3A, we observed a general shift in the distribution of the mRS towards better functional outcomes in favor of AIS over ICH (generalized odds ratio [OR] 1.69, 95% confidence interval [CI] 1.02–2.79), p = 0.04). In particular, the achievement of an excellent functional outcome, i.e. a mRS of 0 to 1, was strikingly more frequent in patients with AIS compared to ICH (n = 23/97 [23.7%] vs n = 8/97 [8.2%], p < 0.01). The results of the ordinal shift analysis remained consistent after additional adjustment for duration from symptom onset to CT with maximum lesion volume and non-aggregated ASPECTS location (OR 1.91, 95% CI 1.09–3.35), p = 0.02). We performed additional subgroup analyses, see Fig. 3B. Better functional outcomes in favor of AIS over ICH were observed in the subgroup of younger patients (age < 75 years, OR: 3.23, 95% CI 1.58–6.59, p < 0.01) and the subgroup of patients with smaller lesion volumes (< 15 mL, OR 4.48, 95% CI 1.85–10.89, p < 0.01). Furthermore, patients with AIS in whom the lesion was lobar in location had slightly more pronounced differences in favorable outcome compared to patients with ICH (any lobar affection, OR 1.88, 95% CI 1.02–3.50, p = 0.045). There were no differences regarding patient sex and the side of stroke lesion, and no subgroup showed an outcome favoring ICH. In both cohorts, a higher lesion volume was correlated with a more severe functional outcome (AIS, r = 0.361; ICH, r = 0.491, both p < 0.001).

### Survival analysis

**Fig. 4:**
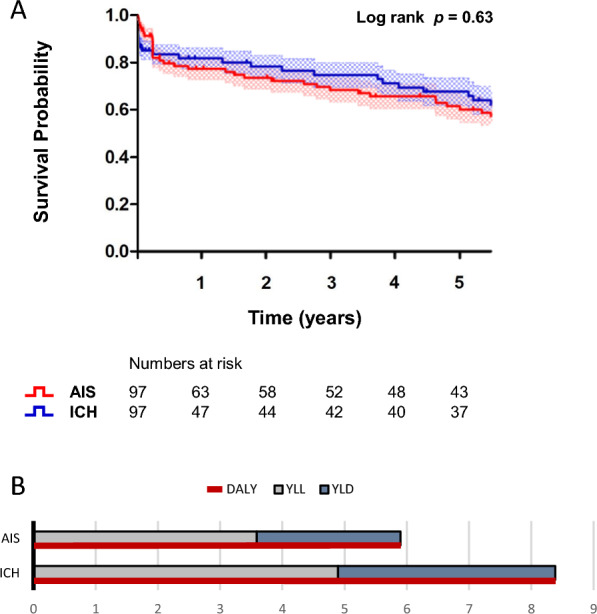
Kaplan Meier survival and DALYs analysis. 4A: Five-year survival probability for patients with AIS (red line) and ICH (blue line). The number of patients at risk is indicated below the x-axis. The mean follow-up duration was 2.6 ± 2.3 years. Crosses indicate censored data, the standard error of the mean is represented by the squared patterns. No significant difference in survival was observed between the groups (log-rank test, p = 0.63). 4B: This figure shows DALY distributions between groups (ICH, 8.39 ± 5.18 vs. AIS, 5.90 ± 4.61, p < 0.001, red line), showing also YLL with ICH, 4.90 ± 3.27 vs. AIS, 3.59 ± 3.08, p < 0.01 and YLD with ICH, 3.49 ± 3.3 vs. AIS, 2.31 ± 2.86, p < 0.01

There was no difference in the rates of in-hospital mortality (AIS, n = 4/97 [4.1%] vs. ICH, n = 8/97 [8.2%], p = 0.23) or mortality at three months (AIS, n = 16/97 [16.5%] vs. ICH, n = 13/97 [13.4%], p = 0.55) between matched patients with AIS and ICH. During a mean follow-up of 2.6 (± 2.3) years, a total of 43 (44.3%) deaths occurred in the AIS group and 38 (39.2%) in the ICH group. The median follow-up period of 2.6 (± 2.3) years is consistent with the expected mortality rates in these populations, with estimates indicating a 5-year survival rate of 41–53% following ICH and 50–64% following AIS [28, 30–32]. Figure 4A shows the cumulative survival over 5 years using Kaplan–Meier estimates and number-at-risk tables to illustrate the distribution of person-time at risk throughout the follow-up period. The survival probability of matched patients with AIS and ICH did not differ over time (log-rank 0.63, hazard ratio = 0.88, 95% CI [0.52–1.50], p = 0.63).

### Burden of disease

The mean number of DALYs was higher in patients with ICH compared to matched patients with AIS (ICH, 8.39 ± 5.18 vs. AIS, 5.90 ± 4.61, p < 0.001), see Figure 4B. Poorer functional outcome resulted in higher number of YLD in patients after ICH compared to matched patients after AIS (ICH, 3.49 ± 3.3 vs. AIS, 2.31 ± 2.86, p < 0.01). The YLL were also higher in patients after ICH (ICH, 4.90 ± 3.27 vs. AIS, 3.59 ± 3.08, p < 0.01). A calculation of the disease burden for the stroke volume showed that the number of DALYs per milliliter parenchymal lesion is greater in the case of ICH than in the case of AIS [DALYs per mL, ICH, 0.31 (0.14–0.81) vs. AIS, 0.24 (0.10–0.41), p = 0.04]. This indicates that the burden of disease per affected stroke volume is relatively greater by 29% in cases of hemorrhagic stroke compared to those of ischemic stroke.

## Discussion

This comparative observational study showed that for the same lesion size and location, patients with ICH have a worse 3-month functional outcome than patients with AIS. However, the long-term survival of the matched cohorts did not differ, but there was a higher overall burden of disease following ICH.

There is a lack of large cohort studies specifically addressing this topic. The study by Toyoda et al. analyzed 135,266 AIS and 36,014 ICH patients over a 20-year period from the Japan Stroke Data Bank, which highlights advances in standardized recanalization therapy for AIS in comparison to ICH [10]. Girgenti et al. compared 300 AIS and 300 ICH patients matched by age, sex, lesion size, location, and admission date, and reported worse long-term outcomes for patients with ICH [11]. However, the imaging-based matching criteria in their study were considerably less precise than those used in our cohort [11]. Specifically, Girgenti et al. did not perform propensity-score matching; instead, each ICH patient was paired with an AIS patient of the same sex and with only approximate similarity in age (± 10 years), lesion volume (± 10 cc), and lesion location (categorized broadly as hemispheric or subcortical without finer ASPECTS-based subdivision) [11].

We observed a more pronounced disparity in functional outcome in lesions of a smaller size, suggesting that the nature of the damage may exert a more considerable effect in such cases. In addition to the initial mechanical or metabolic damage mechanism, a potential underlying reason for this phenomenon might be a more harmful perifocal edema in ICH compared to AIS, a factor that becomes more relevant when the lesion is small. We suggest that if lesions are above a certain size, a critical threshold could be reached in both types of strokes, which in principle makes an unfavorable outcome more likely. Previous studies confirmed that patients with large lesion volumes have a poor prognosis, regardless of whether the cause is an ischemic stroke or an ICH [32, 33]. Clinical imaging studies of perifocal edema have shown a first peak at 2–5 days and a second peak at 2–3 weeks in patients with ICH, whereas in patients with ischemic stroke perilesional edema reaches only one single peak at 3–5 days [23, 34–36]. It has been suggested that the time-delayed edema formation of ICH patients is caused by the breakdown of the blood–brain barrier and numerous experimental studies have shown that this is related to the leakage of blood and its degradation products (e.g. various hemoglobin and iron compounds) and products formed during coagulation (e.g. thrombin) [37–40]. This additional toxic effect by blood degradation products on healthy surrounding tissue could explain why ICH patients were more severely impaired. Furthermore, the discrepancy in functional outcome was more pronounced in younger patients (< 75 years). These patients generally have a higher regeneration potential than older patients, both in ischemic stroke and ICH [41, 42]. However, the observed functional differences might suggest that the processes occurring during ICH disrupt the regeneration of the affected and immediately adjacent brain tissue to a greater extent than in AIS, although the progressive atrophy of the brain with age may help to attenuate the effects of intracranial volume expansion associated with ICH.

Once the critical initial phase has been overcome, it seems reasonable that the 5-year survival rate does not differ. The included patients with moderately sized stroke (AIS, 21 mL, [9–44] [; ICH, 26 mL [8–47]) showed equally high hazards for mortality over time (hazard ratio = 0.88 [0.52–1.50]). The precisely defined parenchymal lesion in patients with comparable clinical characteristics therefore appears to have an impact on functional impairment, but not on long-term survival with these impairments. It is important to note that the inclusion of patients with intraventricular hematoma expansion or the need for surgical intervention might have yielded different mortality rates. However, the present results are consistent with those of larger registry studies, which, after an initial increase in short-term mortality following ICH, did not find any further increased mortality over the subsequent five-year period, nor did they find any association between ICH and subsequent mortality after adjusting for stroke severity [32, 33].

With regard to the burden of disease, prior investigation identified that ICH was overall associated with a mean number of 9.5 DALYs, which was substantially more than 5.9 DALYs reported after severe ischemic stroke [13, 43]. In this selected cohort of patients with stroke lesions only in the MCA territory, we observed similar numbers of and differences in DALYs (ICH, 8.4; AIS, 5.9). Moreover, we provide DALYs per milliliter of parenchymal lesion, and quantify a disease burden weighing 1.29 times more per affected volume after ICH compared to AIS. The inclusion of this population-based epidemiological health measure in the comparison provides an additional quantitative dimension in the analysis of stroke types, combining both survival and functional impairment. Unlike single-time-point functional scores, DALYs capture the long-term impact of stroke on both mortality and quality of life, extending across the patient’s remaining lifespan. As a continuous, interval-scaled metric, they offer greater statistical power for detecting therapeutic effects and allow a more comprehensive assessment of disease burden and healthcare impact, which can guide resource allocation and the prioritization of rehabilitation services [13, 28, 29, 44]. By additionally calculating the DALYs per milliliter of lesion volume, we aim to improve the interpretability of the DALYs in the context of lesion burden.

This study is subject to several limitations. First, we retrospectively analyzed monocentric data from two registries, which reduces data quality and limits generalizability, especially given the lack of external validation or a replication cohort. Different inclusion time periods may introduce treatment-related confounding. Yet, data were collected at the same university center and in overlapping time periods, thus minimizing interrater bias and regional treatment differences. Second, for the matching procedure, we had to simplify the applied ASPECTS segmentation by merging neighboring areas and reducing them from 10 to 5 areas in order to achieve a sufficient sample size for outcome analyses. This may have affected the accuracy of the results, as subtle but clinically relevant location effects may have been obscured. However, the impact appears to be limited, as several adjacent areas are regularly affected above a certain lesion size. The power of the subgroup analysis in cohorts after matching procedure may be limited due to the small sample size. Third, the control imaging following the initial treatment phase was selected as the matching time point assuming the proper infarct demarcation in AIS and lesion stability in ICH. We have not made any statement regarding the extent to which the therapy administered up to this point has influenced the lesions. However, the question addressed here refers to a situation in which the best possible treatment has already been carried out in two different conditions for which therapy cannot be compared in terms of prognostic assessment. It cannot be ruled out that CT does not capture full infarct size or additional small ischemic lesions. Fourth, the concept of DALYs per milliliter of lesion volume might appear overly reductionist; nevertheless, our intention is to address a prognostically relevant gap that could provide an additional and potentially valuable perspective. Finally, despite the screening of comprehensive large cohorts, the number of patients who met the inclusion criteria and remained after the rigorous matching procedure may have been insufficient to detect minor statistical discrepancies or to definitively reject type two errors.

## Conclusions

This study provides a multiparametric analysis of the outcomes in matched patients with similarly sized and localized ischemic and hemorrhagic stroke lesions. ICH was associated with worse functional outcome, particularly in younger patients and smaller lesions, and a higher disease burden per mL, while long-term survival remained comparable to AIS.

## Data Availability

The datasets used and/or analysed during the current study are available from the corresponding author on reasonable request.

## Abbreviations

AIS: Acute ischemic stroke
ASPECTS: Alberta Stroke Program Early CT Score
C: Caudate
CI: Confidence interval
CT: Computer tomography
DALYs: Disability-Adjusted Life Years
e.g.: Exempli gratia
I: Insular cortex
IC: Internal capsule
ICH: Intracerebral hemorrhage
i.e.: Id est
IV: Intravenous
L: Lentiform nucleus
M1: Anterior MCA cortex, which corresponds to the frontal operculum
M2: MCA cortex lateral to the insular ligament, corresponding to the anterior temporal lobe
M3: Posterior MCA cortex, corresponding to the posterior temporal lobe
M4: Anterior MCA territory immediately superior to M1
M5: Lateral MCA territory immediately superior to M2
M6: Posterior MCA territory immediately superior to M3
MCA: Middle cerebral artery
ml: Mililiter
mRS: Modified ranking scale
n: Number
OR: Odds ratio
vs: Versus
YLD: Years Lived with Disability
YLL: Years of Life Lost
